# Conventional Trabeculotomy versus Gonioscopy-Assisted Transluminal Trabeculotomy: A Retrospective Cohort Study

**DOI:** 10.3390/jcm11010046

**Published:** 2021-12-23

**Authors:** Masashi Takata, Hiroto Ishikawa, Tomohiro Ikeda, Fumi Gomi

**Affiliations:** Department of Ophthalmology, Hyogo College of Medicine, Nishinomiya 6638501, Japan; Ohmyeye@gmail.com (H.I.); t-ikeda@hotmail.co.jp (T.I.); fgomi@hyo-med.ac.jp (F.G.)

**Keywords:** glaucoma, conventional trabeculotomy, gonioscopy-assisted transluminal trabeculotomy, Schlemm’s canal

## Abstract

Background: Conventional trabeculotomy (CT) is performed in an ab-externo manner with at most 120 degrees of incision area of Schlemm’s canal (SC). Recently, gonioscopy-assisted transluminal trabeculotomy (GATT), which makes possible a 360-degree incision area of SC in an ab-interno manner, is introduced. The purpose of this study was to compare surgical outcomes for CT and GATT with and without simultaneous phacoemulsification over 24 months and to identify factors associated with surgical success. Results: Patients’ baseline characteristics were not significantly different between two groups. The surgical success rate in CT and GATT with phacoemulsification groups were 40.4% and 96.6% and were significantly higher in the GATT group than in the CT group (*p* < 0.001). However, the surgical success rate in CT and GATT without phacoemulsification groups were 40.8% and 54.2%, and there were no significant differences between two groups without phacoemulsification (*p* = 0.55). Similarly, the postoperative IOP was significantly lower in the GATT group than in the CT group only in eyes with simultaneous phacoemulsification. There were no significant differences in the numbers of glaucoma medications between the two groups. Additional glaucoma surgery was needed in 13.2% and 25.9% of patients in the GATT and CT groups, respectively (*p* = 0.157). The multivariate logistic regression analysis revealed that the surgical success of trabeculotomy was significantly associated with combined phacoemulsification and the type of glaucoma surgery (GATT). Conclusion: Although both groups without phacoemulsification had a similar surgical success and IOP-lowering effect, GATT combined with phacoemulsification had a higher surgical success rate and a greater IOP-lowering effect compared with combined CT and phacoemulsification. Multivariate logistic regression analysis showed that the factors associated with higher surgical success at one year and two years postoperatively were the combined phacoemulsification procedure and the GATT.

## 1. Introduction

Glaucoma is the second leading cause of blindness worldwide [1]. In Japan, the reported prevalence of all forms of glaucoma is 5.0% among individuals aged ≥40 years [2]. Retinal ganglion cell death by apoptosis leads to optic nerve fiber loss, resulting in progressive visual field loss [3]. The only evidence of effective therapy is a reduced intraocular pressure (IOP) by medications and surgical procedures [4]. Commonly performed surgical procedures for glaucoma include trabeculotomy [5,6], trabeculectomy [7], and glaucoma drainage device surgery [8].

Trabeculectomy is very effective for reduction of IOP and is necessary for patients with advanced glaucoma. However, this procedure is associated with a risk of bleb infection, which is seen within five years postoperatively in 2.2% of patients who have undergone trabeculectomy [9]. Moreover, late-onset complications (e.g., hypotony maculopathy, bleb leaks, and avascular bleb) are reported disadvantages of trabeculectomy [10,11].

Trabeculotomy creates an incision of Schlemm’s canal (SC), the main site of resistance to aqueous humor outflow [12]. The efficacy of trabeculotomy lies in its dependence on physiological aqueous humor outflow after the incision of SC, and it is mildly effective for IOP reduction; therefore, from the viewpoint of maximal safety, trabeculotomy is generally performed in patients with early- to middle-phase glaucoma of primary open-angle glaucoma (POAG), steroid-induced glaucoma, exfoliative glaucoma, and pediatric glaucoma [5,13,14].

Conventional trabeculotomy (CT) is performed in an ab-externo manner that causes later conjunctival scarring, which often leads to difficulty in performing additional trabeculectomy. The incision area of SC is maximally 120 degrees using metal U-shaped probes [5]. Recently, there have been many reports that gonioscopy-assisted transluminal trabeculotomy (GATT) is effective for controlling IOP in adult open-angle glaucoma [15,16]. GATT procedures are performed in an ab-interno manner, and GATT enables 360-degree incision area of SC while keeping the conjunctiva intact.

In the present study, we retrospectively examined the clinical effects of these two trabeculotomy techniques, and investigated factors associated with surgical success.

## 2. Materials and Methods

This retrospective study involved patients who had undergone trabeculotomy at Hyogo College of Medicine Hospital from January 2014 to June 2018 without prior glaucoma surgery and could be followed up for ≥3 months. When both eyes were eligible, the first operated eye was selected for the study. All patients had medically uncontrolled glaucoma (IOP ≥ 21 mmHg). The data for the patients who underwent CT from November 2014 to December 2016 were collected, and the data for the patients who underwent GATT from August 2016 to June 2018 were also collected. The study was performed in accordance with the Declaration of Helsinki and with approval from the ethics committee of Hyogo College of Medicine (No. 3781).

Two experienced surgeons (MT and TI) performed CT, and one of them (MT) performed GATT. When the patients were aged >50 years, and/or the ophthalmologist believed that the cataract was a contributing factor in visual loss, we considered performing concurrent phacoemulsification.

CT was performed by modifying the ab-externo trabeculotomy technique described by Harms and Dannheim [17] as follows: (1) A conjunctival incision and a 4 × 4-mm scleral flap of four-fifths thickness was created at the limbus. (2) The external wall of SC was opened over the full width of the scleral flap. (3) Metal U-shaped probes (Nagata’s trabeculotomy probe; Inami, Tokyo, Japan) were inserted into both sides of the flap and rotated 90 degrees into the anterior chamber against the trabecular meshwork. (4) The scleral flap was closed with four 10-0 nylon sutures, and the conjunctival flap was then closed. If necessary, standard phacoemulsification was performed after the CT procedure through the same previously created upper scleral incision.

GATT was performed by modifying the 360-degree suture trabeculotomy technique [18] as follows: (1) Topical 4% lidocaine was applied preoperatively, and a standard sterile procedure was performed. A near-limbal corneal port was created on the temporal side using a 2.2-mm slit knife (Alcon, Fort Worth, TX, USA). After the induction of intracameral anesthesia using preservative-free 1% lidocaine (Sandoz pharma, Tokyo, Japan), the anterior chamber was filled with ophthalmic viscosurgical device (1% Healon^®^) (AMO, Santa Ana, CA, USA). (2) The surgeon used a surgical gonio (Volk, Mentor, OH, USA) for optimal viewing. Approximately 30 degrees of microscope tilt combined with approximately 45 degrees of head tilt was required to achieve an optimal gonioscopic view of the angle. SC was incised through a temporal corneal incision at an angle of 15 degrees on the nasal side using a 20-gauge V-Lance Knife (Alcon, Geneva, Switzerland). (3) The additional oblique port was created using a 20-gauge V-Lance knife to insert 5-0 nylon at the corneal limbus. Next, the rounded tip of a 5-0 nylon suture was inserted into SC using 23-gauge disposable forceps. (4) The suture was pulled out through the same opening after passing the suture tip around the circumference of SC. If the suture stopped advancing into SC, it was removed from the temporal corneal incision site to perform SC incision for the inserted range. Additionally, a suture was inserted into the opposite side of the SC incision site, then pulled out through the tip of the 5-0 nylon suture. (5) The surgeon attempted a 360-degree incision of the SC as long and wide as possible. Standard phacoemulsification was performed following the GATT procedure through the same previously created corneal incision, according to the inclusion criteria. In addition, another side port was created for the insertion of a nuclear divider using a 20-gauge V-Lance knife at the lower corneal limbus during phacoemulsification. Then, OVD was removed from the anterior chamber using a bimanual irrigation-aspiration system, and the corneal incision site was hydrated with balanced salt solution to prevent postoperative hypotony. At the end of the surgery, the subconjunctival injection of 0.44 mg of dexamethasone sodium phosphate (Sandoz pharma, Tokyo, Japan) and 1 mg of gentamicin sulfate (Nichi-iko, Toyama, Japan) was performed. After the surgery, topical 1.5% levofloxacin (Pfizer, New York, NY, USA) was applied four times daily for 2 months. Topical 0.1% betamethasone (Shionogi pharma, Osaka, Japan) was applied four times daily for 2 weeks, and then, Topical 0.1% betamethasone was changed to topical 0.1% fluorometholone (Nitten Pharmaceutical Co., Nagoya, Japan), and it was applied four times daily for 2 months postoperatively. If simultaneous phacoemulsification was performed, topical 0.1% diclofenac was applied three times daily for 1 month postoperatively. The decision of whether glaucoma medication was prescribed depended on the surgeon’s judgement.

We analyzed the ophthalmological data of preoperatively and 1 day, 1 week, and at least 1, 3, 6, 12, and 24 months postoperatively. Parameters evaluated were collected from the medical records: preoperative baseline characteristics included age, sex, type of glaucoma, preoperative IOP, number of glaucoma medications, and lens status; information at the timing of surgery included the extent of SC incision with and without simultaneous cataract surgery; and postoperative records included postoperative IOP, number of glaucoma medications, incidences of hyphema and transient IOP elevation, and presence or absence of additional trabeculectomy. IOP was measured using a Goldmann applanation tonometer (Haag-Streit Diagnostics, Köniz, Switzerland). When counting the number of glaucoma medications, combination glaucoma drops were assigned 2 points, and single glaucoma drops and oral medications were assigned 1 point.

The primary endpoint was the surgical success rate in each surgery as follows: The surgical success was defined as (1) normal range of IOP (>5 to ≤18 mmHg) with or without the use of topical glaucoma medication, (2) a ≥20% reduction of IOP from baseline with or without the use of topical glaucoma medication at two consecutive visits, and (3) no need for further glaucoma surgery. Kaplan–Meier survival analysis was used to evaluate the surgical success rates. The secondary endpoints were the changes in IOP, the number of glaucoma medications, and the medication-free rates in all eyes at each postoperative time point as well as the incidences of postoperative hyphema and transient IOP elevation in the two groups. The presence or absence of hyphema was determined at 1 day postoperatively. A transient postoperative IOP elevation was defined as IOP of ≥25 mmHg that occurred between 1 week and 1 month postoperatively, followed by reduction to ≤21 mmHg.

Continuous variables are presented as mean, standard deviation, and range. Discrete variables are presented as the number and percentage of values in each category. Student’s *t*-test or the Wilcoxon signed-rank test was used to assess group differences in continuous variables, and Fisher’s exact test or Pearson’s χ^2^ test was used to assess group differences in categorical variables. Comparisons between pre- and postoperative IOP and numbers of glaucoma medications at each time points were performed by using one-way ANOVA with Dunnett’s test as post hoc. The intergroup differences in IOP and number of glaucoma medication during the follow-up period were analyzed by analysis of covariance (ANCOVA), correcting for the influence of preoperative IOP and preoperative numbers of glaucoma medications. We used univariate and multivariate logistic regression analysis to identify the factors associated with the surgical success. Based on the results obtained in the univariate analysis, the variables to be analyzed in the multivariate analysis were selected. Analyses were performed with JMP Pro version 14.0.0 (SAS Institute, Cary, NC, USA). Kaplan–Meier survival plots were constructed to assess the long-term survival rates and were compared using the log-rank test. For all analyses, both *p*-values and two-sided 95% confidence intervals are reported for point estimates. Statistical significance was defined as a *p*-value of <0.05. If necessary, a Bonferroni correction was used for multiple/repeated testing.

## 3. Results

### 3.1. Baseline Characteristics

In total, we analyzed 80 eyes of 80 patients. The baseline characteristics are shown in Table 1. In brief, the patients’ mean age was 68.6 ± 13.6 years, and 37 patients were male (46.3%). The patients were separated into two groups: the CT group (27 patients, 27 eyes) and the GATT group (53 patients, 53 eyes). The preoperative MD value of Humphrey^®^ field analyzer (HFA) 30-2 was −7.36 ± 5.45 dB; the MD value was significantly lower in the GATT group (−8.34 ± 5.95 dB) than in the CT group (−5.33 ± 3.43 dB). The preoperative glaucoma subtypes and lens conditions were not different between the two groups. Combined cataract extraction was performed in 43 eyes (53.8%), 13 eyes (48.1%) in the CT group and 30 eyes (56.6%) in the GATT group, and there were no significant differences between the two groups. The mean range of extent of incision in SC in the CT group (117.8 degrees ± 11.3 degrees) was significantly smaller than that in the GATT group (328.3 degrees ± 60.7 degrees). The postoperative observation period was 18.4 ± 7.5 (3–24) months for CT and 20.0 ± 5.8 (6–24) for GATT.

### 3.2. Primary Endpoint: Surgical Success Rate

**Figure 1:**
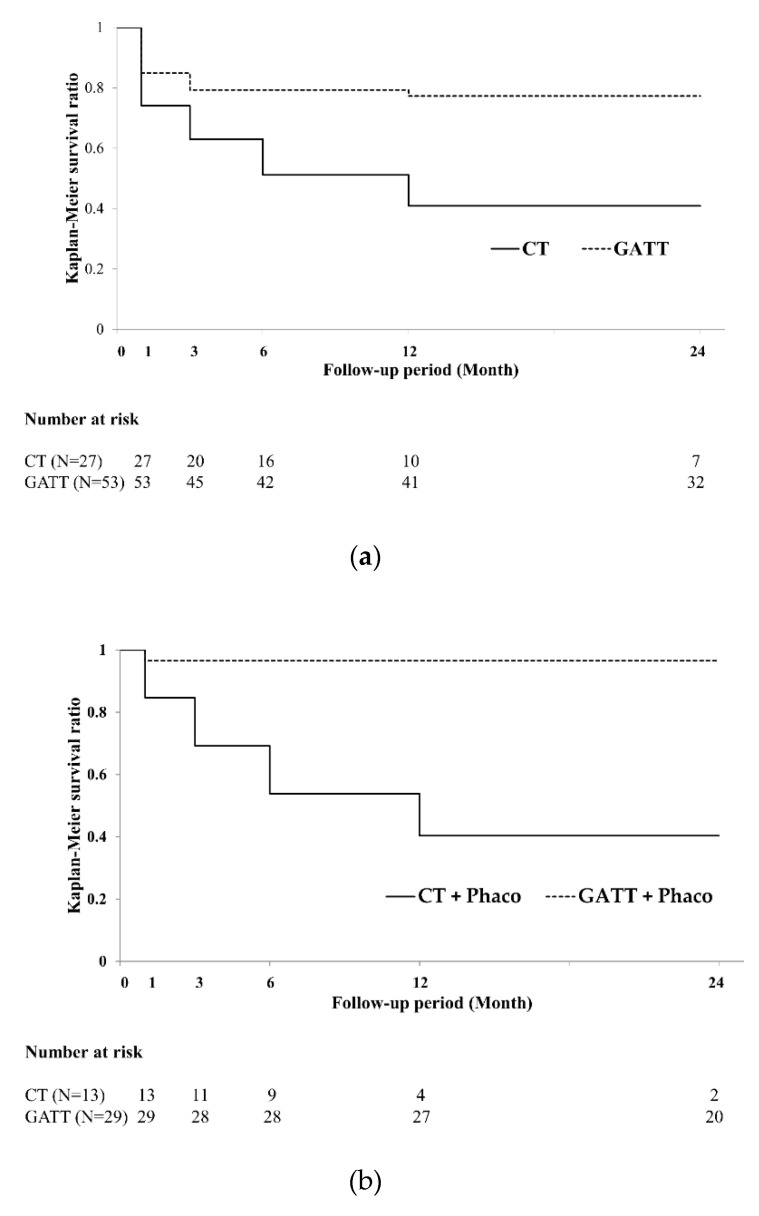

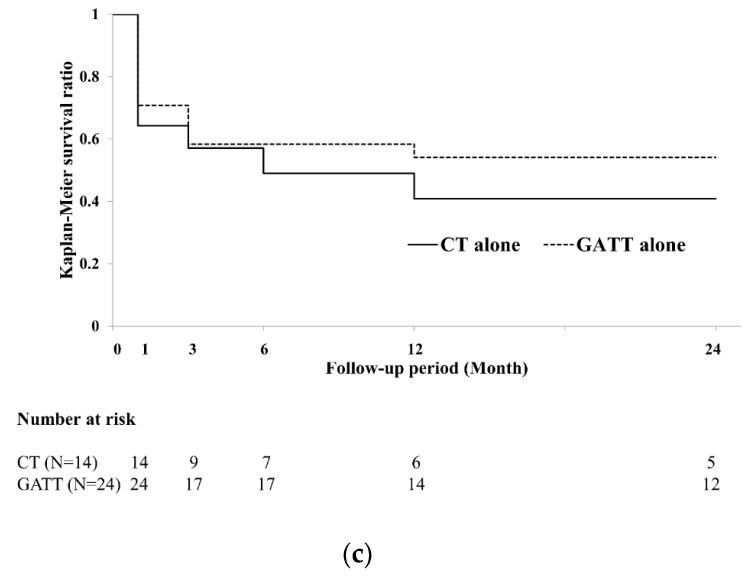
Kaplan–Meier survival curves of surgical success in the CT (solid line) and GATT (dotted line) groups. (**a**) Survival curve of all eyes in the CT and GATT group. Differences between the two groups were significant using the log-rank test (*p* < 0.005). (**b**) Survival curve in eyes that underwent CT or GATT with phacoemulsification. Differences between the two groups were statistically significant using the log-rank test (*p* < 0.001). (**c**) Survival curve in eyes that underwent CT or GATT without phacoemulsification. Differences between the two groups were not statistically significant using the log-rank test (*p* = 0.55). CT, conventional trabeculotomy; GATT, gonioscopy-assisted transluminal trabeculotomy.

The Kaplan–Meier survival curves indicated that the surgical success rate in the CT and GATT groups at 24 months postoperatively were 40.9% and 77.3%, respectively (Figure 1a). The surgical success rates at 24 months postoperatively were significantly higher in the GATT group than in the CT group (log-rank test, *p* < 0.005). We also analyzed data from two groups with and without phacoemulsification. The surgical success rate in the CT and the GATT with phacoemulsification at 24 months postoperatively were 40.4% and 96.6%, respectively (Figure 1b). The surgical success rates at 24 months postoperatively were significantly higher in the GATT with phacoemulsification group than in the CT with phacoemulsification group (log-rank test, *p* < 0.001). The surgical success rate in the CT without phacoemulsification and GATT without phacoemulsification groups at 24 months postoperatively were 40.8% and 54.2%, respectively (Figure 1c), and there were no significant differences between both CT and GATT groups without phacoemulsification at 24 months postoperatively (log-rank test, *p* = 0.169 and 0.55, respectively).

**Figure 2:**
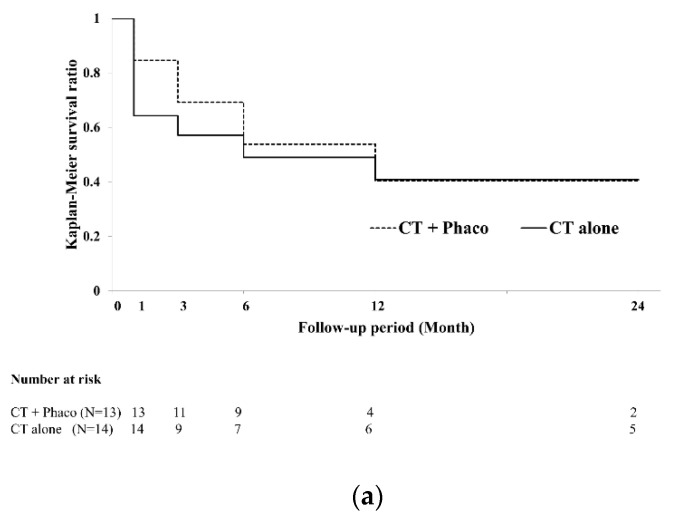

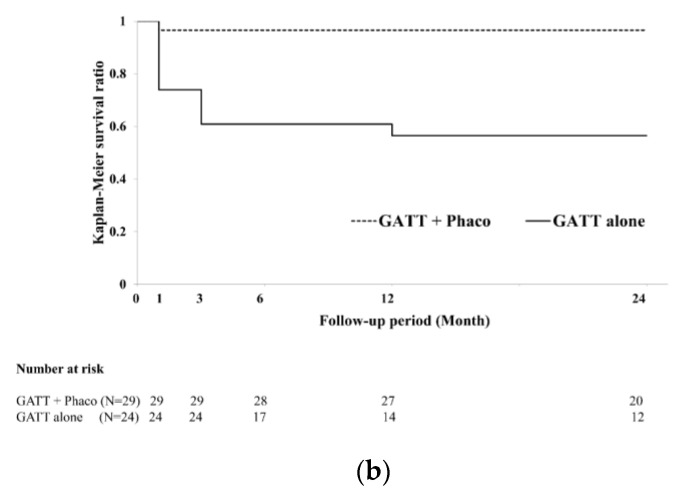
Kaplan–Meier survival curves of surgical success in the with phacoemulsification (solid line) and without phacoemulsification (dotted line) groups. (**a**) Survival curve in eyes that underwent CT with or without phacoemulsification. (**b**) Survival curve in eyes that underwent GATT with or without phacoemulsification. The GATT combined with phacoemulsification showed significantly higher surgical success rate than that without phacoemulsification (log-rank test, *p* < 0.001). CT, conventional trabeculotomy; GATT, gonioscopy-assisted transluminal trabeculotomy.

Then, to investigate the effect combined phacoemulsification in CT and GATT, we analyzed the surgical success rate in each group with and without phacoemulsification. The surgical success rate in CT with and without phacoemulsification at 24 months postoperatively were 40.4% and 40.8% (Figure 2a). There were no significant differences between CT with and without phacoemulsification group (log-rank test, *p* = 0.793), The surgical success rate in GATT with and without phacoemulsification at 24 months postoperatively were 96.6% and 54.2% (Figure 2b). The surgical success rates were significantly higher in the GATT with phacoemulsification group than in the GATT without phacoemulsification group (log-rank test, *p* < 0.001).

### 3.3. Secondary Endpoints: Other Postoperative Outcomes

The secondary endpoints were changes in IOP, number of glaucoma medications, medication-free rates among patients, and rates of additional glaucoma surgery in all eyes at each postoperative time point as well as the incidences of postoperative hyphema and transient IOP elevation between the CT and GATT groups.

#### 3.3.1. Changes in IOP

The postoperative IOP and the changes in IOP in each group are shown in Table 2. In brief, the postoperative IOP was significantly lower than the preoperative IOP in both CT and GATT groups (*p* = 0.053 at one day in CT, *p* < 0.001 at other each time point in CT and GATT). Moreover, the IOP was significantly lower in the GATT group than in the CT group at one day and 6, 12, and 24 months postoperatively (*p* < 0.001, *p* < 0.001, *p* = 0.024, and *p* = 0.020, respectively). Additionally, the IOP was significantly lower in the GATT group with phacoemulsification than in the CT group, with phacoemulsification at 1 day and 1, 3, 6, 12, and 24 months (*p* = 0.010, *p* = 0.013, *p* = 0.004, *p* = 0.008, *p* < 0.001, and *p* = 0.004, respectively). However, there were no significant differences in postoperative IOP between the CT and GATT groups without phacoemulsification at one week and 1, 3, 6, 12 and 24 months postoperatively (*p* = 0.932, *p* = 0.792, *p* = 0.831, *p* = 0.089, *p* = 0.535, and *p* = 0.584, respectively), whereas there were differences at one day postoperatively (*p* = 0.011). Additional glaucoma surgery was required in seven eyes (25.9%) in the CT group and seven eyes (13.2%) in the GATT group. There was no significant difference in the rates between the two groups (*p* = 0.16).

#### 3.3.2. Changes in the Number of Glaucoma Medications

The changes in the number of glaucoma medications are shown in Table 3. In brief, the postoperative number of glaucoma medications was significantly lower than the preoperative number in both CT and GATT groups (*p* = 0.0129 and *p* = 0.0048 at 12 and 24 months in CT, *p* < 0.001 at other each time point in CT and GATT). The numbers of glaucoma medications in the GATT group were significantly lower than the numbers in the CT group at 12 months postoperatively (*p* = 0.018). Moreover, there were no significant differences in the numbers of glaucoma medications between both CT and GATT groups with and without phacoemulsification at all postoperative points.

#### 3.3.3. Medication-Free Rates among Patients

The rates of being free of medication are shown in Table 4. In brief, these rates in the GATT group were significantly higher than those in the CT group at three and six months postoperatively (*p* = 0.016, and *p* = 0.039). However, there was no significant difference in the rates between the groups at 12 and 24 months. Regardless of simultaneous phacoemulsification, there were no significant differences in medication-free rates between the two groups.

#### 3.3.4. Incidences of Hyphema and Transient IOP Elevation

Hyphema occurred in 12 of 27 eyes (44.4%) in the CT group and in 31 of 53 eyes (58.5%) in the GATT group. Transient IOP elevation occurred in six of 27 eyes (22.2%) in the CT group and in eight of 53 eyes (15.1%) in the GATT group. There were no significant differences in the rates of hyphema and transient IOP elevation between the two groups (*p* = 0.233, and *p* = 0.428).

### 3.4. Factors Associated with the Surgical Success

The factors associated with the surgical success were investigated using univariate and multivariate logistic regression analysis. Univariate and multivariate logistic regression analysis at one year and two years postoperatively are shown in Table 5 and Table 6. The type of glaucoma surgery (GATT vs. CT), combined phacoemulsification, and the occurrence of IOP spike were the factors selected using the univariate logistic regression were analyzed by multivariate logistic regression. The multivariate logistic regression analysis revealed that the surgical success at one year and two years postoperatively was significantly associated with combined phacoemulsification (odds rate, 5.39, 95% confidence interval, 1.62–17.88, *p* = 0.006; odds rate, 4.44, 95% confidence interval, 1.30–15.20, *p* = 0.018, respectively) and the type of glaucoma surgery (GATT or CT) (odds rate, 4.30, 95% confidence interval, 1.37–13.43, *p* = 0.012; odds rate, 4.25, 95% confidence interval, 1.26–14.38, *p* = 0.020, respectively).

## 4. Discussion

Most areas of aqueous humor outflow resistance are thought to be associated with the para-SC connective tissue involving the trabecular meshwork [12]. Therefore, trabeculotomy, which incises the SC, is presumed to reduce IOP by reducing aqueous humor outflow resistance. In this study, we retrospectively compared CT and GATT, two methods of trabeculotomy with different range of SC incision. Although both CT and GATT reduced the IOP postoperatively, GATT had a higher surgical success rate than CT, and the combined phacoemulsification was a significant factor to achieve higher success in the GATT group. However, among patients with no phacoemulsification, there were no significant differences in surgical success rate between the CT and GATT groups. In addition, in terms of IOP reduction, GATT with phacoemulsification had a stronger effect on IOP reduction than CT at 24 months postoperatively, but the effect of IOP reduction was similar between GATT without phacoemulsification and CT without phacoemulsification. On the other hand, there were no significant differences in the numbers of glaucoma medications between both CT and GATT groups with phacoemulsification or between CT and GATT groups without phacoemulsification. Moreover, multivariate logistic regression analysis showed that the factors associated with surgical success at one year and two years postoperatively were the presence of combined phacoemulsification and the type of glaucoma surgery (GATT).

The main differences between CT and GATT are the extent of the incision of SC and with and without a conjunctival incision. CT involves a maximal 120-degree incision [5], whereas GATT can be extended to a 360-degree incision [6,18]. In an enucleated human eye, it was reported that encircling the SC incision (360 degrees) resulted in a 75% loss of outflow resistance, and a 120-degree incision in SC had an 85% IOP reduction compared to a 360-degree incision in SC [19]. However, clinical results associated with the incision range during trabeculotomy are controversial. Chin et al. [20] compared the 18 months’ surgical effects of trabeculotomy between CT and 360-degree trabeculotomy ab-externo by metal probe and reported that the postoperative IOP and number of glaucoma medications were significantly lower in patients undergoing 360-degree trabeculotomy ab externo than for CT. These finding are consistent with our findings in the present study. However, Manabe et al. [21] reported that no correlation was present between IOP reduction at 12 months postoperatively and the extent of SC incision. Otori et al. [22] also reported that there were no significant differences in the surgical outcomes and postoperative complication between CT and GATT.

In our study, the multivariate analysis revealed that GATT affected surgical success rates positively. It is reported that the outflow resistance is not consistent with the area of the trabecular meshwork [23], and there could be “segmental outflow” (i.e., division between high and low outflow) [23,24]. A recent report suggests more numbers of the collector channels could be present in the high-outflow segment [25]. The collector channels are thought to localize at the nasal SC much more than at the temporal SC [26,27]. Thus, the proportion of collector channel openings at the site of the SC incision may also be related to the effect of IOP reduction and the surgical success rate. Although GATT can make a wider SC incision than CT, the extent of SC incision alone cannot explain how it is that GATT is a good prognostic factor for surgical success rates. GATT may entail another mechanism that reduces IOP in addition to attenuation of outflow resistance by SC incision, and it is considered that further research, including histological examination, is necessary.

In this study, the multivariate analysis revealed that the presence or absence of combined phacoemulsification had the greatest effect on the surgical success. It is commonly reported that cataract extraction can reduce IOP [28]. Cataract extraction induces greater anterior chamber depth, which might affect the zonules, ciliary body, and angle structures [29]. This interaction with the ciliary body might prevent SC narrowing [30] and facilitate the outflow of aqueous humor. Interestingly, our study showed that the combined cataract extraction and GATT was more effective than combined cataract extraction and CT. Bektas et al. [31] reported that the simultaneous phacoemulsification surgery was a positive prognostic factor on the surgical success rate of the GATT. On the contrary, Shinmei et al. [32] reported that there were no significant differences in IOP changes and numbers of glaucoma medications between the groups of a modified 360-degree suture trabeculotomy ab externo with and without simultaneous cataract surgery technique. More research is needed to clarify the mechanism underlying the differences in outcomes between groups with and without phacoemulsification.

The early postoperative complications, such as hyphema or transient IOP elevation (IOP spikes), were observed in both groups. Because the extent of SC incision was wider in the GATT group than in the CT group, the incidences of hyphema and IOP spikes were expected to be higher in the GATT group than in the CT group. However, there were no significant differences between the two groups in the present study. Alnahrawy et al. [33] reported that no correlation was present between the amount of hyphema and the extent of SC incision. Furthermore, there was no significant difference in the incidence of IOP spikes between the two groups, consistent with previous findings that the extent of SC incision does not influence transient IOP elevation [21]. Therefore, early postoperative complications were considered similar after the two procedures.

The present study had several limitations. First, this was a retrospective study; it was not prospective or randomized. Second, the follow-up duration was limited to 24 months; a longer follow-up may deliver different results. Third, the sample size of the group was small and involving eyes with different types of glaucoma and the severity, which may have affected the surgical outcomes. Fourth, the proportion of patients who completed 24 months of follow-up was reduced by approximately half compared with the initial number of included patients due to additional glaucoma surgery, loss to follow-up, and referrals to other hospitals in both groups. This patient dropout might have affected the outcomes. Further prospective studies with larger sample sizes and longer follow-up times will be necessary to fully evaluate the efficacy of the two procedures.

Our retrospective study based on real-world data showed that both GATT alone and CT alone had a similar effect of the IOP reduction and the long-term cumulative probability of surgical clinical success, but GATT with phacoemulsification had a greater and longer effect of IOP reduction and the cumulative probability of surgical clinical success compared with CT. In addition, the combined phacoemulsification procedure and the GATT were factors associated with higher surgical success. Combined phacoemulsification with GATT might enhance the possibility of surgical clinical success.

## Figures and Tables

**Table 1:** Patients’ characteristics.

	Total	CT	GATT	*p*-Value
	80 Eyes	27 Eyes	53 Eyes	
Age (mean ± SD) years	68.6 ± 13.6	65.8 ± 12.8	70.0 ± 13.7	0.06
Sex (male), *n* (%)	37 (46.3)	11 (40.7)	26 (49.1)	0.48
Preoperative IOP (mean ± SD) (mmHg)	30.7 ± 8.63	30.3 ± 8.38	30.96 ± 8.74	0.76
Preoperative number of glaucoma medications	4.04 ± 1.02	4.11 ± 0.99	4.00 ± 1.03	0.59
Preoperative MD of HFA 30-2 (dB) (mean ± SD)	−7.36 ± 5.45	−5.33 ± 3.43	−8.34 ± 5.95	0.03
Type of glaucoma				0.55
POAG, *n* (%)	31 (38.75)	10 (37.04)	21 (39.62)	
Exfoliative, *n* (%)	28 (35.0)	8 (29.63)	20 (37.74)	
Steroid-induced, *n* (%)	14 (17.5)	7 (25.93)	7 (13.21)	
Others, *n* (%)	7 (8.75)	2 (7.40)	5 (9.43)	
Lens status				
Phakic, *n* (%)	53 (66.25)	17 (63.0)	35 (66.0)	0.79
Combined cataract extraction, *n* (%)	43 (53.8)	13 (48.1)	30 (56.6)	0.47
Extent of incision in SC, mean ± SD (degrees)	257.3 ± 111.3	117.8 ± 11.3	328.3 ± 60.7	* <0.001
Follow-up period (mean ± SD, range) (months)	19.5 ± 6.5 (3–24)	18.4 ± 7.5 (3–24)	20.0 ± 5.8 (6–24)	0.41

CT, conventional trabeculotomy; GATT, gonioscopy-assisted transluminal trabeculotomy; SD, standard deviation; MD, mean deviation; HFA, Humphrey field analyzer; SC, Schlemm’s canal. By using a Bonferroni correction for multiple comparisons, * *p* < 0.005 was defined as statistical significance.

**Table 2:** Pre- and postoperative IOP in the CT and GATT with and without phacoemulsification.

	Preoperative	1 Day	1 Week	1 Month	3 Months	6 Months	12 Months	24 Months
CT, mean (median) ± SD (mmHg)	30.04 (27) ± 8.38 (*n* = 27)	26.78 (26) ± 9.14 (*n* = 27)	18.00 (18) ± 4.19 (*n* = 27)	16.07 (16) ± 3.54 (*n* = 27)	16.33 (17) ± 3.47 (*n* = 27)	17.38 (17.5) ± 2.97 (*n* = 24)	18.15 (18) ± 4.33 (*n* = 20)	17.75 (18) ± 3.42 (*n* = 16)
GATT, mean (median) ± SD (mmHg)	30.96 (28) ± 8.73 (*n* = 53)	18.26 (16) ± 9.01 (*n* = 53)	17.51 (16) ± 7.94 (*n* = 53)	14.85 (14) ± 6.30 (*n* = 52)	14.76 (14) ± 5.27 (*n* = 50)	14.31 (14) ± 3.65 (*n* = 49)	14.93 (14) ± 6.57 (*n* = 45)	15.37 (16) ± 3.54 (*n* = 35)
*p*-Value	NA	* <0.001	0.717	0.376	0.179	* <0.001	* 0.024	* 0.020
CT with phacoemulsification, mean (median) ± SD (mmHg)	27.69 (26) ± 5.03 (*n* = 13)	28.00 (26) ± 8.38 (*n* = 13)	18.31 (16) ± 3.93 (*n* = 13)	16.31 (16) ± 2.52 (*n* = 13)	16.08 (17) ± 1.90 (*n* = 13)	17.17 (17.5) ± 2.82 (*n* = 12)	17.25 (16.5) ± 3.27 (*n* = 8)	19.14 (20) ± 2.42 (*n* = 7)
GATT with phacoemulsification, mean (median) ± SD (mmHg)	28.60 (26.5) ± 7.14 (*n* = 30)	19.33 (16) ± 9.28 (*n* = 30)	17.30 (15.5) ± 7.40 (*n* = 30)	13.67 (14) ± 3.06 (*n* = 30)	13.30 (13) ± 2.97 (*n* = 30)	13.80 (13) ± 3.39 (*n* = 30)	13.25 (13.5) ± 2.06 (*n* = 28)	15.05 (16) ± 3.39 (*n* = 21)
*p*-Value	NA	* 0.010	0.615	* 0.013	* 0.004	* 0.008	* <0.001	* 0.004
CT without phacoemulsification, mean (median) ± SD (mmHg)	32.21 (30) ± 10.11 (*n* = 14)	25.64 (26) ± 9.66 (*n* = 14)	17.71 (18) ± 4.40 (*n* = 14)	15.86 (16.5) ± 4.26 (*n* = 14)	16.57 (17) ± 4.45 (*n* = 14)	17.58 (17.5) ± 3.09 (*n* = 12)	18.75 (19) ± 5.37 (*n* = 12)	16.67 (16) ± 3.68 (*n* = 9)
GATT without phacoemulsification, mean (median) ± SD (mmHg)	34.04 (30) ± 9.62 (*n* = 23)	16.87 (14) ± 8.46 (*n* = 23)	17.78 (16) ± 8.58 (*n* = 23)	16.45 (13.5) ± 8.75 (*n* = 22)	16.95 (16) ± 6.94 (*n* = 20)	15.11 (14) ± 3.91 (*n* = 19)	17.71 (16) ± 9.74 (*n* = 17)	15.86 (16) ± 3.70 (*n* = 14)
*p*-Value	NA	* 0.011	0.932	0.792	0.831	0.089	0.535	0.584

CT, conventional trabeculotomy; GATT, gonioscopy-assisted transluminal trabeculotomy; SD, standard deviation; NA, not applicable; * *p* < 0.05.

**Table 3:** Pre- and postoperative number of glaucoma medications in the CT and GATT with and without phacoemulsification.

	Preoperative	1 Week	1 Month	3 Months	6 Months	12 Months	24 Months
CT, mean (median) ± SD	4.11 (4) ± 0.99 (*n* = 27)	1.93 (2) ± 1.27 (*n* = 27)	2.11 (2) ± 1.37 (*n* = 27)	2.37 (2) ± 1.22 (*n* = 27)	2.50 (2) ± 1.32 (*n* = 24)	2.80 (3) ± 1.57 (*n* = 20)	2.88 (3) ± 1.54 (*n* = 16)
GATT, mean (median) ± SD	4.00 (4) ± 1.03 (*n* = 53)	1.42 (1) ± 1.51 (*n* = 53)	1.68 (2) ± 1.55 (*n* = 53)	1.72 (2) ± 1.58 (*n* = 50)	1.82 (2) ± 1.61 (*n* = 49)	1.87 (2) ± 1.61 (*n* = 45)	1.97 (2) ± 1.73 (*n* = 35)
*p*-Value	NA	0.139	0.222	0.070	0.081	* 0.018	0.095
CT with phacoemulsification, mean (median) ± SD	3.69 (4) ± 1.07 (*n* = 13)	1.85 (2) ± 1.35 (*n* = 13)	2.23 (2) ± 1.19 (*n* = 13)	2.31 (2) ± 1.26 (*n* = 13)	2.42 (2) ± 1.38 (*n* = 12)	2.63 (3.5) ± 1.65 (*n* = 8)	3.14 (4) ± 1.55 (*n* = 7)
GATT with phacoemulsification, mean (median) ± SD	4.03 (4) ± 0.75 (*n* = 30)	1.60 (1.5) ± 1.52 (*n* = 30)	1.57 (1) ± 1.56 (*n* = 30)	1.57 (1) ± 1.50 (*n* = 30)	1.73 (1.5) ± 1.63 (*n* = 30)	1.79 (1.5) ± 1.61 (*n* = 28)	2.00 (2) ± 1.80 (*n* = 21)
*p*-Value	NA	0.766	0.225	0.164	0.249	0.227	0.206
CT without phacoemulsification, mean (median) ± SD	4.50 (4.5) ± 0.73 (*n* = 14)	2.00 (2) ± 1.20 (*n* = 14)	2.00 (2) ± 1.51 (*n* = 14)	2.43 (2) ± 1.18 (*n* = 14)	2.58 (2) ± 1.26 (*n* = 12)	2.92 (3) ± 1.50 (*n* = 12)	2.67 (2) ± 1.49 (*n* = 9)
GATT without phacoemulsification, mean (median) ± SD	3.96 (4) ± 1.30 (*n* = 23)	1.17 (0) ± 1.46 (*n* = 23)	1.77 (2) ± 1.54 (*n* = 22)	1.91 (2) ± 1.65 (*n* = 20)	1.95 (2) ± 1.57 (*n* = 19)	2.00 (2) ± 1.61 (*n* = 17)	1.93 (2) ± 1.62 (*n* = 14)
*p*-Value	NA	0.165	0.688	0.356	0.202	0.09	0.306

CT, conventional trabeculotomy; GATT, gonioscopy-assisted transluminal trabeculotomy; SD, standard deviation, * *p* < 0.05.

**Table 4:** Changes in medication-free rates.

Time Point	Total			With Phacoemulsification			Without Phacoemulsification		
	CT (%)	GATT (%)	*p*-Value	CT (%)	GATT (%)	*p*-Value	CT (%)	GATT (%)	*p*-Value
1 month	22.20 (6/27)	37.70 (20/53)	0.161	15.38 (2/13)	40.00 (12/30)	0.108	28.57 (4/14)	34.78 (8/23)	0.493
3 months	11.10 (3/27)	36.00 (18/50)	0.016	15.38 (2/13)	36.67 (11/30)	0.151	7.14 (1/14)	35.00 (7/20)	0.067
6 months	12.50 (3/24)	34.70 (17/49)	0.039	16.67 (2/12)	36.67 (11/30)	0.187	8.33 (1/12)	31.58 (6/19)	0.143
12 months	15.00 (3/20)	31.11 (14/45)	0.144	25.00 (2/8)	32.14 (9/28)	0.532	8.33 (1/12)	29.41 (5/17)	0.182
24 months	12.50 (2/16)	29.41 (10/34)	0.171	14.29 (1/7)	30.00 (6/20)	0.393	11.11 (1/9)	28.57 (4/14)	0.327

CT, conventional trabeculotomy; GATT, gonioscopy-assisted transluminal trabeculotomy. By using a Bonferroni correction for multiple comparisons.

**Table 5:** Factors associated with the surgical success at 1 year postoperatively.

Variables	Univariate Analysis		Multivariate Analysis	
	OR (95% CI)	*p*-Value	OR (95% CI)	*p*-Value
GATT (vs. CT)	4.81 (1.66–13.99)	* 0.004	4.30 (1.37–13.43)	* 0.012
Combined phacoemulsification	4.43 (1.55–12.64)	* 0.005	5.39 (1.62–17.88)	* 0.006
Occurrence of IOP spike	0.44 (0.13–1.55)	0.202	3.11 (0.76–12.78)	0.115
Age	1.02 (0.98–1.07)	0.297		
Preoperative IOP	0.99 (0.93–1.04)	0.669		
Preoperative glaucoma medications	1.20 (0.73–1.98)	0.466		
Occurrence of hyphema	0.89 (0.33–2.34)	0.810		
POAG	1.93 (0.68–5.46)	0.216		
PEG	0.66 (0.24–1.83)	0.422		

OR, odds rate; CI, confidence interval; CT, conventional trabeculotomy; GATT, gonioscopy-assisted transluminal trabeculotomy; POAG, primary open-angle glaucoma; PEG, pseudoexfoliative glaucoma, * *p* < 0.05.

**Table 6:** Factors associated with the surgical success at 2 years postoperatively.

Variables	Univariate Analysis		Multivariate Analysis	
	OR (95% CI)	*p*-Value	OR (95% CI)	*p*-Value
GATT (vs. CT)	4.95 (1.59–15.38)	* 0.006	4.25 (1.26–14.38)	* 0.020
Combined phacoemulsification	3.70 (1.29–11.47)	* 0.014	4.44 (1.30–15.20)	* 0.018
Occurrence of IOP spike	0.26 (0.06–1.17)	0.080	3.44 (0.71–16.64)	0.124
Age	1.02 (0.98–1.06)	0.292		
Preoperative IOP	0.98 (0.93–1.04)	0.570		
Preoperative glaucoma medications	1.05 (0.63–1.73)	0.858		
Occurrence of hyphema	0.75 (0.27–2.04)	0.569		
POAG	2.20 (0.77–6.78)	0.142		
PEG	0.59 (0.20–1.70)	0.329		

OR, odds rate; CI, confidence interval; CT, conventional trabeculotomy; GATT, gonioscopy-assisted transluminal trabeculotomy; POAG, primary open-angle glaucoma; PEG, pseudoexfoliative glaucoma, * *p* < 0.05.

## Data Availability

The datasets analyzed during the current study are available from the corresponding author on reasonable request.
